# Reasons for Cancellation of Cases on the Day of Surgery–A Prospective Study

**Published:** 2009-02

**Authors:** Rakesh Garg, Anju R Bhalotra, Poonam Bhadoria, Nishkarsh Gupta, Raktima Anand

**Affiliations:** 1 Senior Resident,AIIMS, Maulana Azad Medical College and associated Lok Nayak Hospital, New Delhi; 2 AssociateProfessor, Maulana Azad Medical College and associated Lok Nayak Hospital, New Delhi; 3 Professor, Maulana Azad Medical College and associated Lok Nayak Hospital, New Delhi; 4 Junior Consultant,MaxHospital, Maulana Azad Medical College and associated Lok Nayak Hospital, New Delhi; 5 Director,Professor andHead, Maulana Azad Medical College and associated Lok Nayak Hospital, New Delhi

**Keywords:** Cancellation, Operation room, Postponement cases

## Abstract

**Summary:**

Late cancellation of scheduled operations is a major cause of inefficient use of operating-room time and a waste of resources. We studied elective operating theatre bookings in general surgical discipline. On the day of surgery the intended list was noted and a list of cancellations with the reason was noted by the attending anaesthesiologist.

1590 patients were scheduled for elective surgical procedures in 458 operation rooms. 30.3 % patients were cancelled on the day of surgery. Of these, 59.7% were cancelled due to lack of availability of theatre time, 10.8% were cancelled because of medical reasons and 16.2% did not turned up on the day of surgery. In 5.4% patients, surgery was cancelled by surgeons due to a change in the surgical plan, 3.7% were cancelled because of administrative reasons, and 4.2% patients were postponed because of miscellaneous reasons.

We believe that many of the on-the-day surgery cancellations of elective surgery were potentially avoidable. We observed that cancellations due to lack of theatre time were not only a scheduling problem but were mainly caused by surgeons underestimating the timeneeded for the operation. The requirement of the instruments necessary for scheduled surgical list should be discussed a day prior to planned OR list and arranged. The non-availability of the surgeon should be informed in time so that another case is substituted in that slot. All patients who have met PACU discharge criteria must be discharged promptly to prevent delay in shifting out of the operated patient. Day care patients should be counseled adequately to report on time. Computerized scheduling should be utilized to create a realistic elective schedule. Audit should be carried out at regular intervals to find out the effective functioning of the operation theatre.

## Introduction

Major hospitals invest considerable resources in maintaining operating suites and having anaesthesiologists, surgeons and theatre staff available on an agreed schedule. However, unanticipated cancellation of scheduled operations at the last minute, even on the morning of surgery is of concern. In some cases, patients have even been prepared for theatre and staff is assembled and expecting to operate. Late cancellation of scheduled operations is a major cause of inefficient use of operating-room time and a waste of resources. It is also potentially stressful with depressing effects and costly to the patient in terms of working days lost and disruption of daily life^1^,^2^.

We undertook a prospective study on the day surgery cancellations in a government hospital in New Delhi with the aim to find out the causes of cancellation of cases scheduled on the day of surgery and to suggest measures for optimum utilization of operating room (OR)time.

## Methods

At our hospital, all patients are evaluated in the preanaesthesia clinic well before surgery and obtain PAC clearance prior to being posted for surgery. Difficult cases (anticipated long surgeries or patients with poor general condition or comorbidities or difficult airways) are shown to the concerned anaesthesiologist a day prior to surgery. The operating list is prepared by the surgeons, and sent to OR by afternoon.

We studied elective operating theatre bookings in general surgical discipline at a large tertiary government hospital in Delhi (1542 resourced beds, 16 operating theatres) for 6 months. A cancellation on the day of intended surgery was defined as any operation that was either scheduled on the final theatre list for that day (generated at 15:00 hours on the previous day) or was subsequently added to the list and that was not performed on that day. On the day of surgery the intended list was noted and a list of cancellations with the reason for cancellation was noted by the attending anaesthesiologist.

## Results

1590 patients were scheduled for elective surgical procedures in 458 operation rooms during the study period. 47.7% patients were male and rest being females. 28% of the total surgical procedures were planned laparoscopically.

Of 1590, 482 (30.3 %) patients were cancelled on the day of surgery. 288 out of 482 (59.7%) were cancelled due to lack of availability of theatre time; 52 out of 482 (10.8%) were cancelled because of medical reasons and 78 out of 482 patients (16.2%) did not turn up on the day of surgery. In 26 out of 482 (5.4%) patients, surgery was cancelled by surgeons due to a change in the surgical plan; 18 out of 482 (3.7%) were cancelled because of administrative reasons (autoclaved instruments/linens not available, instrument not available); 20 out of 482 (4.2%) patients were postponed because of miscellaneous reasons (no availability of senior surgeon for the case, ICU bed/ventilator, adequate blood products and refusal of consent by patient)(Table 1).

**Table 1:** Reasons for Cancellation of Surgical Cases (n=480)

Reasons of cancellation	Cancellation%
Lack of operating room time	59.7%
Medical Reasons of the patient	10.8%
Patient did not turned up	16.2%
Change in surgical plan	5.4%
Administrative reasons(autoclaved Instruments/ linens not available, instrument not available)	3.7%
Miscellaneous Reasons (non availability of senior surgeon for the case, ICU bed/ventilator, adequate blood products and refusal of consent by patient)	4.2%

## Discussion

The decision to postpone surgery in a patient after admission for surgery has psychological, social and economic implications, and is not only based on clinical considerations. The reported rates for day-of-surgery cancellation rates vary widely among institutions from 10-40 %. We found that 30.3 % of all scheduled elective operations in general surgery were cancelled on the day of surgery. Fischer reported that almost 90% of operating room (OR) cancellations are day-of-surgery cancellations^3^.

We believe that many of the on-the-day surgery cancellations of elective surgery were potentially avoidable in our audit.

Jonnalagadda et al reported the reasons for cancellation of scheduled routine and emergency cases as non-availability of beds in the recovery room (15%), improper preoperative patient preparation (13%), patient not showing up (9%), and unavailability of staff (19%). They also mentioned that public patients were cancelled more frequently than private patients^4^.

Schofield et al in their study of cancellation of intended surgery at a major hospital in Australia reported 941 (11.9%) cancellations out of 7913 theatre sessions. The reasons included no bed available (18.9%), run out of theatre time (16.1%), patient non-arrival (10.5%), patient unfit (9.2%), and cancelled by patient or relatives (8.2%)^1^.

Vinukondaiah et al cited the major reasons for cancellation of cases in the general surgery OR to be lack of operating time (65.2%), emergency surgery during the elective list (13.9%), and lack of fitness (11.3%)^5^. In our institute cancellation of elective cases due to emergency cases was not a problem because of the presence of a dedicated emergency OR. But sometime, the senior surgeon is called to emergency OR for help thereby delaying or wasting routine OR time leading to the postponement of an elective case.

Windokun et al reported that only 38% of the booked surgery was performed and the reasons for such cancellation included ‘surgeons did not show up’ (62%), ‘surgery postponed by surgeons’ (18%) and ‘patient ill prepared for surgery’ (10%)^6^.

In our study non-availability of OR time was the most common reason. We observed that cancellations due to lack of theatre time were not only a scheduling problem but were mainly caused by surgeons underestimating the time needed for the operation. Surgeons generally add more patients to the OT list to reduce the waiting list and in anticipation of any unexpected cancellations. An analysis in USA examining 56,000 cases retrospectively found that 31% of lists were predictably overbooked^1^. Moreover, unforeseen anaesthetic or surgical problems may delay the planned list. The time taken for a particular surgery also depends on the skill of operating surgeon. Less experienced surgeons and trainees often take more than the expected time. For some surgeries the total duration exceeded the usual surgical time due to an unexpected surgical complication, juniors being taught and allowed to do the surgery especially for laparoscopic procedures, unavailability of sterilized instruments, and technical problems in instruments.

Hsiao et al suggested having of dedicated minimally invasive surgery suites to save time in transporting of equipments and thus optimizing utilization of OR time^7^. In our audit too, one of the reasons for delay in the start of surgery was because of time required to arrange laparoscopic equipments as sometimes it was being used in the other OR.

Ogden et al reported OR time over run in 27% and reasons mentioned were improper utilization of OR time and undue delay when junior surgeons/anaesthesiologist performed the cases^8^. Pandit et al concluded that over running OR lists were the commonest cause of the cancellation of cases on the day of surgery (50% lists were overbooked and 50% over ran their scheduled time)^9^.

Late start of the OR due to absence of staff has also been reported to lead to underutilization of OR time leading to cancellation of the cases^10^. Weinbroum et al reported that 15% of the OR time was wasted due to inappropriately prepared patients, unavailability of surgeons, insufficient OR staff, congestion of PACU, and delay in the transport to the OR^11^.

The anaesthesia time was variable among patients even for similar surgeries. This was probably because of patient physical status, anaesthetist's expertise and technical problems. However Hussain reported that only 8% of all cancellation of cases on the day of surgery was anaesthesia related^12^.

An accurate real time based schedule should be made considering the expected duration of surgery, the availability of staff, equipments and correct instruments for a smooth running OR.

Medical cancellations are generally presumed to be another reason of cancellations. Because cancellations caused by medical problems are especially upsetting for patients and can be more contentious for members of the medical staff these cancellations may be more memorable than other types of cancellations. Inadequate preoperative medical optimization was another important reason for cancellation of cases in our study. The major reasons were hypertension, recent onset respiratory tract infections, uncontrolled diabetes and an acute onset cardiovascular abnormality. Some cancellations due to failure to comply with the preoperative orders and the development of a medical illness can be minimized by a preoperative visit by the anaesthesiologist and the surgeon a day prior to scheduled surgery^3^.

Providing more beds or quarantining beds for surgical patients is one component of an improved system but will be insufficient unless all sources of problems receive attention. Robb et al reported that 31% of the cancelled case for the elective procedures were post-poned because of “No Bed’ status^13^.

Last-minute cancellation due to failure of a patient to present is especially difficult to resolve. It may be due to the patient's last minute doubts and fears. Efforts should be made to improve patient communication and facilitate their compliance with scheduled procedures. Paschoal reported that 54.3% cases of the total cancelled cases were due to absenteeism of the patient because of unawareness of the date of surgery, clinical problems like respiratory tract infections and social/economical reasons^14^.

Disruptions in the power supply has been mentioned as one of the causes of delay in the operation in third world countries^15^.

Cancellations may occur due to scheduling errors, inadequate preoperative evaluation, inadequate patient preparation, lack of surgical linen, equipment shortage, non-availability of the trained staff etc. This is because of the lack of coordination of different departments involved in the functioning of operating rooms and lack of efficient management of operating theatre floor. These reasons are avoidable if proper administrative measures are taken. These cancellations may lead to dissatisfied patients and can be quite costly. Surgical cancellations could be regarded as adverse events and monitored routinely in hospital clinical incident monitoring systems.

Overlapping induction, i.e., induction of anaesthesia with an additional team while the previous patientis still in the OR has been analyzed and reported to increase the OR productivity by decreasing the nonoperative timeby 45.6%^16^. However this requires additional staff and equipments thus increasing the over-all cost. However, we can save OR time by inserting epidural catheters and peripheral and central intravenous access in the side room prior to shifting the patient to the OR while the previous patientis still in the OR.

All staff concerned with the operating schedule should be punctual to ensure cases are done at planned time. The operating list should be made judiciously. Meticulous care and proper planning must be taken to complete the OR list daily. It is the duty of the theatre-in-charge in consultation with surgeons to ensure that there is no wastage of operating timen or is there over-crowding of the list leading to postponement of surgery. Any postponement of surgery should be justified.

The requirement of the instruments/drugs/other equipment necessary for scheduled surgical list should be discussed among surgeon, staff nurse and the anaesthesiologist a day prior to planned OR list. The non-availability of the surgeon should be informed in time so that another case is substituted in that slot. All patients who have met PACU discharge criteria must be discharged promptly to prevent delay in shifting out of the operated patient. Day care patients should be counseled adequately to report on time. Computerized scheduling should be utilized to create a realistic elective schedule. Audit should be carried out at regular intervals to find out the effective functioning of the OR.
